# Investigating the Characteristics of Pelvic Ultrasound in Girls With Their First Menstrual Cycle: A Two‐Center Cross‐Sectional Study

**DOI:** 10.1002/hsr2.70961

**Published:** 2025-06-30

**Authors:** Masoud Mahdavi Rashed, Peyman Eshraghi, Ehsan Hassannejad, Mostafa Jafarpour

**Affiliations:** ^1^ Department of Radiology, Faculty of Medicine Mashhad University of Medical Sciences Mashhad Iran; ^2^ Department of Pediatrics, Faculty of Medicine Mashhad University of Medical Sciences Mashhad Iran; ^3^ Department of Radiology, School of Medicine Birjand University of Medical Sciences Birjand Iran

**Keywords:** menarche, puberty, ultrasound, uterus

## Abstract

**Background and Aims:**

One of the important modalities in the case of precocious puberty diagnosis is pelvic ultrasonography. This method provides information about the internal uterus and ovaries. The aim of this study is to assess pelvic sonography parameters in Iranian girls who experience menarche.

**Method:**

This cross‐sectional study was conducted in the radiology department of Akbar and Dr. Sheikh hospitals in 2022. The onset of puberty symptoms should not have been before 8 years old. A pediatric endocrinologist visited all the participants to ensure a normal puberty process. An expert radiologist conducted ultrasound parameters measurements. A predesigned questionnaire was used to assess the demographic and anthropometric data, and all data were analyzed.

**Result:**

Fifty girls were enrolled in the study with a median age of 11 years old. The mean of the longitudinal diameter of the uterus was 61.8 ± 7.59 mm, the mean of the transverse diameter of the uterus was 35.91 ± 5.01 mm, the mean of the anteroposterior diameter of the uterus was 24.76 ± 4.81 mm, and the mean of the uterus volume was 30.04 ± 11.84 cm³. The association of weight, height, and BMI with different sonography measurements was assessed. No significant association between these findings and BMI was found (*p* > 0.05); however, a significant correlation was found between height and anteroposterior diameter of the uterus (*p* = 0.015). Linear regression analysis showed a positive relationship between height and anteroposterior diameter of the uterus (*p* = 0.027).

**Conclusion:**

The ultrasonography measurements may differ among different populations, and studies should be conducted on different ethnicities. Our results were comparable to those of other studies; however, the age differed, and the studies did not focus on menarche time.

## 1 Introduction

Puberty is the developmental transition from childhood to adolescence and adulthood. This term may be posed for different aspects of becoming mature; however, it is physiologically termed as the condition that prepares a person for breeding. The condition usually starts between the ages of 9–13 years old [1, 2]. The process involves emotional and physical changes; however, the physical changes are much bolder. The physiology that underlies puberty is associated with many interconnected neuronal and hormonal changes; however, there is a changing course in the phenotype of the girl during this time [3].

Normal puberty in girls usually starts with pubarche, which results from a physiologic change termed adrenarche [4]. This usually happens at 6–8 years old ages. Pubarche is followed by breast enlargement during the age of 9 or 10, which is termed thelarche. The final stage, which is defined as the first menstrual period in a female adolescent, is usually menarche, which is triggered between 10 and 16 ages [5]. Puberty in boys generally begins between the ages of 9 and 14, characterized by an initial increase in testicular size, followed by growth of the penis, the appearance of pubic hair, and voice changes. Onset of the pubertal growth spurt precedes in girls compared to boys. In both males and females, pubarche can precede the activation of the gonads [6, 7]. Regarding this, precocious puberty (PP) in girls is the start of puberty symptoms before the age of 8 years old [8].

The gold standard diagnosis of precocious puberty is the GnRH test [9]. However, as the method is expensive, barely accessible, and hard to use, many radiology modalities are investigated for the diagnosis of PP. In addition, a matter of concern is the inconsistent application of gonadotropin cutoff values in the diagnosis of central precocious puberty [10]. One of the important modalities in the case of PP diagnosis is pelvic ultrasonography. This method provides information about internal genitalia, including the uterus and ovaries [3].

Concerning uterine and ovarian dimensions in the diagnosis of precocious puberty, although standardization is lacking, some measurements have demonstrated clinical utility [11]. However, many studies in the literature are from decades ago [12, 13, 14, 15, 16, 17]. Also, the role of ethnicity should not be ignored [18].

Research indicates a rise on average ovarian volume from 0.7 mL at age 2–7.7 mL at age 20, followed by a decline to approximately 2.8 mL at menopause [19].

A comprehensive understanding of ovarian growth patterns during childhood is lacking, with no established consensus on normal morphologic characteristics and measurements within the pediatric age range [17].

Limited knowledge exists concerning the standard ovarian morphology in prepubescent individuals, as the prepubertal uterus exhibits a thin structure while its body is similar in size to the cervix. Moreover, despite the common sonographic observation of cystic ovarian structures, their classification is characterized by confusion and a lack of uniformity [20].

In pediatric and adolescent populations, pelvic ultrasonography represents a valuable diagnostic tool due to its reliability, noninvasive nature, and reproducibility. In cases of suspected precocious puberty in girls, an ultrasound is almost invariably requested by the physician. The low levels of perivisceral fat in children make them exceptionally well‐suited for ultrasonographic examination. Repeated examinations are possible due to the absence of irradiation. The aforementioned factors contribute to its increasing utilization in the therapeutic management of a range of pediatric and adolescent endocrine and gynecological disorders, including, but not limited to, hirsutism, precocious puberty, and thelarche. The evaluation of these disorders requires a clear distinction between normal and pathological conditions in light of the significant morphological alterations in pelvic organs that occur during puberty. Therefore, normative data regarding uterine dimensions and ovarian volume across various ages are crucial [21, 22].

For clinical use, normal value ranges are needed to distinguish abnormal measurements accurately. Patient age is expected to influence the variation in such ranges. A significant portion of the pelvic ultrasound data from healthy children is decades old, potentially reflecting technological and epidemiological advancements [18].

Therefore, this study aims to assess pelvic sonography parameters in Iranian girls who experience menarche.

## 2 Materials and Methods

### 2.1 Participants and Design of the Study

This cross‐sectional study was conducted in the radiology department of Akbar and Dr. Sheikh hospitals, affiliated with the Mashhad University of Medical Sciences, in 2022. Fifty girls, aged 9–15 years old, were enrolled in the study. The onset of puberty symptoms should not be before 8 years old. Moreover, secondary diseases that may affect normal puberty should be detected through laboratory screening of FSH, LH, estradiol, prolactin, testosterone, hydroxy progesterone, TSH, T4, FBS, AST, ALT, Alk‐p, BUN, and creatinine. The bleeding disorders were also ruled out. A pediatric endocrinologist visited all the participants to ensure a normal puberty process.

### 2.2 Basic Data

A predesigned questionnaire was used to assess some of the demographic and anthropometric data of the patients. These data included age, weight, height, and body mass index (BMI). A stadiometer was used for height measurement. Moreover, the weight was evaluated using a digital scale. BMI was calculated according to the following formula:

$$\text{BMI}\;=\text{Weight}\;\left(\text{kg}\right){/\text{Height}}^{2}\left(m^{2}\right).$$

### 2.3 Pelvic Sonography

All ultrasound measurements were conducted by an expert radiologist using a sonography device of the Samsung model WS80. The parameters measured in pelvic sonography included longitudinal, anteroposterior (AP), and transverse diameters of the uterus. Also, fundus to cervix ratio (FCR), endometrial thickness, number, and size of ovarian follicles were evaluated. Moreover, the longitudinal and AP diameters of the uterus were measured in the mid‐sagittal view of the uterus and its transverse diameter in the axial section. Finally, the ovarian and uterus volumes were measured using the following formula:

Length×Width×Height×0.523.

### 2.4 Ethics

All the participants' guardians were provided with written informed consent. The data of the enrolled sample was anonymized and coded to be kept top secret. All the steps of this study were in accordance with Helsinki's declaration and were confirmed by the Ethics Committee of the Mashhad University of Medical Sciences (Ethics code: IR.MUMS.MEDICAL.REC.1400.162).

### 2.5 Analysis

All the analyses were accomplished using SPSS version 23. The mean and standard deviation, or median and interquartile range, of demographic, anthropometric, and sonographic data were calculated. Moreover, the association of different sonography findings with age, weight, and BMI was assessed using Pearson's test. A *p* value less than 0.05 was considered significant.

## 3 Result

Fifty girls, with a median age of 11, were enrolled in the study. Table 1 shows demographic and anthropometric data of enrolled participants, including age, weight, height, and BMI.

**Table 1:** Demographic and anthropometric data of enrolled participants.

Parameters	Mean/median (IQR)
Age (years; median [IQR])	11 (12–10.45)
Weight (kg; median [IQR])	45 (50–41.75)
Height (m; mean ± SD)	1.47 ± 0.057
BMI (kg/m^2^; median; IQR])	20.85 (22.81–19.13)

Table 2 shows the mean and standard deviation of the uterus's diameters, including the longitudinal, transverse, and anteroposterior diameters and volume.

**Table 2:** Mean and standard deviation diameters of the uterus.

Parameters	Mean	Standard deviation	Maximum	Minimum
Longitudinal diameter of the uterus (mm)	61.8	7.59	77.9	42.6
Transverse diameter of the uterus (mm)	35.91	5.01	49	27.8
Anteroposterior diameter of the uterus (mm)	24.76	4.81	35	15
Uterus volume (cm^3^)	30.04	11.84	61	12.6

Table 3 shows the median and interquartile range of the uterus and ovaries.

**Table 3:** The median and interquartile range of the uterus and ovaries.

Parameters	Median	Interquartile range
Fondus to cervix ratio (FCR)	2	(2–2)
Endometrial thickness (mm)	7.20	(10.30–6.27)
Right ovary volume (cm^3^)	5.15	(6.45–4.26)
Left ovary volume (cm^3^)	5	(6.87–3.77)
Number of follicles in right ovary	5	(6.25–4)
Number of follicles in left ovary	4	(6–3)
Maximum diameter of follicle in right ovary	8	(10.27–6.90)
Maximum diameter of follicle in left ovary	9	(11.67–6.77)

The association of weight, height, and BMI with different sonography measurements was assessed using the Pearson test. No significant association between these findings and BMI was found (*p* > 0.05); however, a significant correlation was found between height and the AP diameter of the uterus (*p* = 0.015). Linear regression analysis showed a positive relationship between height and the AP diameter of the uterus (*β* = 0.0313; 95% CI = [48.49–3.065]; *p* = 0.027).

Patients were divided into two groups by height: (gruoup1: 1.38–1.50 m) and (group2: 1.51–1.63 m). Table 4 shows the mean and standard deviation of uterine diameters for the two groups.

**Table 4:** The mean and standard deviation of uterine diameters for the two groups divided by height.

Parameters	Group	Mean	Standard deviation
Longitudinal diameter of the uterus (mm)	1	61.92	6.9
	2	61.79	9.3
Transverse diameter of the uterus (mm)	1	34.98	4.45
	2	38.30	5.73
Anteroposterior diameter of the uterus (mm)	1	23.67	4.75
	2	27.57	3.86
Uterus volume (cm^3^)	1	27.88	10.57
	2	35.61	13.47

## 4 Discussion

Ultrasonography of the pelvis, especially internal genitalia, plays an important role in the life of a female. During childhood puberty problems, during childbearing assessments, and the suspicion of gynecology diseases, sonography is the modality of choice for the assessment [23]. The development of the uterus starts a long journey from the 6 weeks of fetal age to adolescence and adulthood. The absence of anti‐müllerian hormone and testosterone results in the differentiation of Wolffian and Müllerian ducts to the female genitalia [24, 25]. The organ continues to grow with a peak in puberty age. Moreover, there will be other changes during the childbearing period. Sonography plays an important role in all of these maturation parts, as it can provide live images with low cost and no ionizing radiation. Even sonography can provide valuable data on the blood flow of the organ. With this regard, this is the modality of choice in genital evaluation during puberty [3, 26].

Therefore, it is important to find the standard ovary and uterine measurements to distinguish abnormal conditions. Gilligan et al. [18] assessed the ultrasonographic measurements of the ovary and uterine from infancy to childhood and adolescence. They assessed a considerable sample of girls in this regard. In the case of endometrial thickness, they reported a mean thickness of 2.7, 3.8, and 5.6 mm in 10‐, 11‐, and 12‐year‐old girls. Moreover, at age 12, the mean uterine, left, and right ovary volumes were 31.6, 4.9, and 6.2 cm^3^, respectively. Also, they reported a longitudinal uterine diameter of 6.5 cm and a transverse diameter of 3.5 cm. We assessed 50 girls with a median age of 11 years old who experienced their menarche. We found an endometrial thickness of 7.20 mm, uterine longitudinal diameter of 6.18 cm, uterine transverse diameter of 3.59 cm, uterine volume of 30.04 cm^3^, left ovary volume of 5 cm^3^ and right ovary volume of 5.15 cm^3^. Still, unlike the study by Gilligan et al. [18], we focused on those girls who experienced their first‐period cycle, and the differences may be due to this fact. However, our results in girls with a median age of 11 were similar to 12 years old girls in Gilligan et al. [18] study. Similar to our findings, Gilligan et al. [18] reported that the right ovary is larger than the left ovary in size. The role of ethnicity should not be forgotten, and part of the age differences can be due to this fact.

Dixit et al. [27] conducted another similar study in Indian girls. They proposed similar volumetric results compared to our study at age 12–13 years old. The uterine length, mean ovarian volume, and fondus to cervix ratio (FCR) were 5.47 cm, 3.02 cm^3^, and 1.46, respectively. They also reported that the amounts increase as the tanner stage gets higher. In our study, uterine longitudinal diameter, right ovarian volume, left ovarian volume, and FCR were 6.18 cm, 5.15 cm^3^, 5 cm^3^, and 2, respectively. However, these results were achieved in girls with a median age of 11 years old and not 12–13 years old girls. Again, the differences in different populations should be addressed.

Ersen et al. [28] studied volumetric assessments of the internal genitalia of Turkish girls aged 6–16 years old using ultrasound examination. At the age range of 12–13 years old and 13‐14 years old, the uterine volumes were 14.45 cm^3^ and 25.47 cm^3^. Furthermore, the ovarian volumes were 5.14 cm^3^ and 4.57 cm^3^ in 12‐13‐ and 13‐14‐years old girls. Interestingly, our results regarding ovarian volume were similar to those of 12‐13‐year‐old girls in the Ersen et al. [28] study and, in the case of uterine volume, were similar to those of 13–14 years old girls in their study. Ersen et al. [28] also proposed an FCR of 1.8 in 14–15‐year‐old girls, which was close to the results of our study.

The ultrasonography measurements may differ among different populations, and studies should be conducted on different ethnicities. We conducted a study among Iranian girls, which is less addressed, and thus, this can be mentioned as a strength of our work. However, the sample size of our study, due to the presence of low resources, was not very large. Thus, it should be advised that other studies with higher sample sizes be conducted in other populations. This study is limited by the inherent challenges of ultrasound, which, while convenient for pelvic organ assessment, particularly in pediatric patients, yields measurements that are not always consistently reproducible or accurate. Still, many studies concentrated on the age ranges and not the fact of the first menstrual time. Studies should be conducted to measure the ovarian and uterus sizes at this time point.

## 5 Conclusion

We conducted a study on girls with their menarche to find normal ovarian and uterus parameters. We found that girls with a median age of 11 years old had their first uterus bleeding time. At this point, uterine, left, and right ovary volumes were 30.04, 5, and 5.15 cm^3^, respectively. We also found a uterus longitudinal length of 6.18 cm, a transversal length of 3.59 cm, and a FCR of 2. These results were comparable to other studies; however, the age differed, and studies did not focus on the menarche time. Further studies are needed to complete these results and further application as normal values to differentiate abnormal conditions.

## Author Contributions

**Masoud Mahdavi Rashed:** conceptualization, investigation, writing – review and editing, writing – original draft, supervision, methodology, project administration. **Peyman Eshraghi:** conceptualization, investigation, writing – original draft, writing – review and editing, methodology. **Ehsan Hassannejad:** conceptualization, writing – original draft, investigation, writing – review and editing, methodology, data curation. **Mostafa Jafarpour:** conceptualization, writing – original draft, investigation, writing – review and editing, methodology, supervision, data curation, project administration.

## Ethics Statement

The study design followed the Declaration of Helsinki and was approved by the Ethics Committee of the Mashhad University of Medical Sciences (Ethics code: IR.MUMS.MEDICAL.REC.1400.162). Informed consent was obtained from all individual participants.

## Conflicts of Interest

The authors declare no conflict of interest.

## Transparency Statement

The lead author Mostafa Jafarpour affirms that this manuscript is an honest, accurate, and transparent account of the study being reported; that no important aspects of the study have been omitted; and that any discrepancies from the study as planned (and, if relevant, registered) have been explained.

## Data Availability

The data and materials supporting this study's findings are available from the corresponding author on request.

## References

[1] R. M. Siervogel , E. W. Demerath , C. Schubert , et al., “Puberty and Body Composition,” supplement, Hormone Research in Paediatrics 60, no. Suppl. 1 (2003): 36–45. 10.1159/000071224 12955016

[2] C. Kuhn , M. Johnson , A. Thomae , et al., “The Emergence of Gonadal Hormone Influences on Dopaminergic Function During Puberty,” Hormones and Behavior 58, no. 1 (2010): 122–137. 19900453 10.1016/j.yhbeh.2009.10.015 PMC2883625

[3] S. Arekhi , A. Omranzadeh , and M. M. Rashed , “Radiology Approach of Precocious Puberty: A Review on Different Available Imaging Modalities,” International Journal of Pediatrics 11, no. 3 (2023): 17495‐513.

[4] R. L. Rosenfield , “Normal and Premature Adrenarche,” Endocrine Reviews 42, no. 6 (2021): 783–814. 33788946 10.1210/endrev/bnab009 PMC8599200

[5] T. Varimo , H. Huttunen , P. J. Miettinen , et al., “Precocious Puberty or Premature Thelarche: Analysis of a Large Patient Series in a Single Tertiary Center With Special Emphasis on 6‐to 8‐Year‐Old Girls,” Frontiers in Endocrinology 8 (2017): 213. 28878739 10.3389/fendo.2017.00213 PMC5572337

[6] A. Papadimitriou and G. P. Chrousos , “Reconsidering the Sex Differences in the Incidence of Pubertal Disorders,” Hormone and Metabolic Research 37, no. 11 (2005): 708–710. 16308841 10.1055/s-2005-870586

[7] I. Atta , T. M. Laghari , Y. N. Khan , S. W. Lone , M. Ibrahim , and J. Raza , “Precocious Puberty in Children,” Journal of the College of Physicians and Surgeons‐‐Pakistan: JCPSP 25, no. 2 (2015): 124–128. 25703757

[8] E. A. Eugster , “Update on Precocious Puberty in Girls,” Journal of Pediatric and Adolescent Gynecology 32, no. 5 (2019): 455–459. 31158483 10.1016/j.jpag.2019.05.011

[9] A. C. Latronico , V. N. Brito , and J.‐C. Carel , “Causes, Diagnosis, and Treatment of Central Precocious Puberty,” Lancet Diabetes & endocrinology 4, no. 3 (2016): 265–274. 26852255 10.1016/S2213-8587(15)00380-0

[10] S. N. Ab Rahim , J. Omar , and T. S. Tuan Ismail , “Gonadotropin‐Releasing Hormone Stimulation Test and Diagnostic Cutoff in Precocious Puberty: A Mini Review,” Annals of Pediatric Endocrinology & Metabolism 25, no. 3 (2020): 152–155. 32871650 10.6065/apem.2040004.002 PMC7538306

[11] L. Sessa , G. Rotunno , G. Sodero , et al., “Predictive Value of Transabdominal Pelvic Ultrasonography for the Diagnosis of Central Precocious Puberty: A Single‐Center Observational Retrospective Study,” Clinical Pediatric Endocrinology 33, no. 4 (2024): 199–206. 39359668 10.1297/cpe.2024-0025 PMC11442698

[12] N. A. Bridges , A. Cooke , M. J. R. Healy , P. C. Hindmarsh , and C. G. D. Brook , “Standards for Ovarian Volume in Childhood and Puberty,” Fertility and Sterility 60, no. 3 (1993): 456–460. 8375526

[13] H. L. Cohen , M. A. Shapiro , F. S. Mandel , and M. L. Shapiro , “Normal Ovaries In Neonates and Infants: A Sonographic Study of 77 Patients 1 Day to 24 Months Old,” American Journal of Roentgenology 160, no. 3 (1993): 583–586. 8430559 10.2214/ajr.160.3.8430559

[14] I. Griffin , T. Cole , K. Duncan , A. Hollman , and M. Donaldson , “Pelvic Ultrasound Measurements in Normal Girls,” Acta Paediatrica 84, no. 5 (1995): 536–543. 7633150 10.1111/j.1651-2227.1995.tb13689.x

[15] H. P. Haber and E. I. Mayer , “Ultrasound Evaluation of Uterine and Ovarian Size From Birth to Puberty,” Pediatric Radiology 24 (1994): 11–13. 8008485 10.1007/BF02017650

[16] S. A. Ivarsson , K. O. Nilsson , and P. H. Persson , “Ultrasonography of the Pelvic Organs in Prepubertal and Postpubertal Girls,” Archives of Disease in Childhood 58, no. 5 (1983): 352–354. 6859914 10.1136/adc.58.5.352 PMC1627855

[17] L. F. Orsini , S. Salardi , G. Pilu , L. Bovicelli , and E. Cacciari , “Pelvic Organs in Premenarcheal Girls: Real‐Time Ultrasonography,” Radiology 153, no. 1 (1984): 113–116. 6473771 10.1148/radiology.153.1.6473771

[18] L. A. Gilligan , A. T. Trout , J. G. Schuster , et al., “Normative Values for Ultrasound Measurements of the Female Pelvic Organs Throughout Childhood and Adolescence,” Pediatric Radiology 49 (2019): 1042–1050. 31093723 10.1007/s00247-019-04419-z

[19] S. A. Soliman , M. A. Algatheradi , A. M. Alqahtani , et al., “Ovarian Volume of Saudi Children and Adolescents in the Southern Region Based on Ultrasound Imaging,” Cureus 15, no. 6 (2023). 10.7759/cureus.40147 PMC10329512 37425524

[20] L. D. Herter , E. Golendziner , J. A. M. Flores , E. Becker , and P. M. Spritzer , “Ovarian and Uterine Sonography in Healthy Girls Between 1 and 13 Years Old: Correlation of Findings With Age and Pubertal Status,” American Journal of Roentgenology 178, no. 6 (2002): 1531–1536. 12034633 10.2214/ajr.178.6.1781531

[21] A. Seth , A. Aggarwal , K. Sandesh , R. S. Solanki , S. Aneja , and G. Kumar , “Pelvic Ultrasonography in Pubertal Girls,” Indian Journal of Pediatrics 69 (2002): 869–872. 12450296 10.1007/BF02723710

[22] A. S. Eksioglu , S. Yilmaz , S. Cetinkaya , G. Cinar , Y. T. Yildiz , and Z. Aycan , “Value of Pelvic Sonography in the Diagnosis of Various Forms of Precocious Puberty in Girls,” Journal of Clinical Ultrasound 41, no. 2 (2013): 84–93. 23124596 10.1002/jcu.22004

[23] V. Talarico , M. B. Rodio , A. Viscomi , E. Galea , M. C. Galati , and G. Raiola , “The Role of Pelvic Ultrasound for the Diagnosis and Management of Central Precocious Puberty: An Update,” Acta bio‐medica: Atenei Parmensis 92, no. 5 (2021): 2021480. 10.23750/abm.v92i5.12295 PMC8689311 34738554

[24] K. Holm , E. Mosfeldt Laursen , V. Brocks , and J. Müller , “Pubertal Maturation of the Internal Genitalia: An Ultrasound Evaluation of 166 Healthy Girls,” Ultrasound in Obstetrics & Gynecology 6, no. 3 (1995): 175–181. 8521066 10.1046/j.1469-0705.1995.06030175.x

[25] N. Josso and R. A. Rey , “What Does AMH Tell us in Pediatric Disorders of Sex Development?,” Frontiers in Endocrinology 11 (2020): 619. 33013698 10.3389/fendo.2020.00619 PMC7506080

[26] B. Yuan , Y.‐L. Pi , Y.‐N. Zhang , P. Xing , H.‐M. Chong , and H.‐F. Zhang , “A Diagnostic Model of Idiopathic Central Precocious Puberty Based on Transrectal Pelvic Ultrasound and Basal Gonadotropin Levels,” Journal of International Medical Research 48, no. 8 (2020): 0300060520935278. 32762408 10.1177/0300060520935278 PMC7416140

[27] M. Dixit , V. Jaiswal , Y. Usmani , and R. Chaudhary , “Uterine and Ovarian Parameters in Healthy North Indian Girls From 5 to 16 Years by Ultrasonography,” Pediatric Endocrinology Diabetes and Metabolism 27, no. 1 (2021): 37–41. 33599435 10.5114/pedm.2020.103112 PMC10227480

[28] A. Ersen , H. Onal , D. Yıldırım , and E. Adal , “Ovarian and Uterine Ultrasonography and Relation to Puberty in Healthy Girls Between 6 and 16 Years in the Turkish Population: A Cross‐Sectional Study,” Journal of Pediatric Endocrinology and Metabolism 25, no. 5/6 (2012): 447–451. 22876537 10.1515/jpem-2012-0014

